# Aqueous cytokine levels are associated with reduced macular thickness after intravitreal ranibizumab for diabetic macular edema

**DOI:** 10.1371/journal.pone.0174340

**Published:** 2017-03-27

**Authors:** Tomoyasu Shiraya, Satoshi Kato, Fumiyuki Araki, Takashi Ueta, Tempei Miyaji, Takuhiro Yamaguchi

**Affiliations:** 1 Department of Ophthalmology, Graduate School of Medicine, The University of Tokyo, Tokyo, Japan; 2 Department of Clinical Trial Data Management, Graduate School of Medicine, The University of Tokyo, Tokyo, Japan; 3 Division of Biostatistics, Tohoku University Graduate School of Medicine, Sendai, Japan; Massachusetts Eye & Ear Infirmary, Harvard Medical School, UNITED STATES

## Abstract

**Purpose:**

It is controversial whether the administration of anti-vascular endothelial growth factor drugs for diabetic macular edema (DME) affects intraocular inflammatory cytokines. In this study, we measured cytokine concentration in aqueous humor before and after intravitreal injection of ranibizumab (IVR). The aim was to determine changes in cytokine concentration and their effects on DME reduction.

**Methods:**

Twelve patients (13 eyes) with DME received two IVR (0.5 mg) with a 1 month interval, and a total of 26 aqueous humor samples were obtained. Macular thickness was measured with an optical coherence tomography (OCT) using thickness-map mode with an Early Treatment Diabetic Retinopathy Study (ETDRS) 9-zone grid that was divided into two zones: a central circle with a diameter of 1 mm (zone1); and an outer circle with a diameter of 6 mm (zone2).

**Results:**

The concentration of eotaxin-1 in aqueous humor samples decreased significantly after IVR. Baseline cytokine concentration was associated with IVR-induced DME reduction. In zone1, higher baseline concentration of interferon-induced protein (IP)-10, and in zone 2, higher baseline concentration of granulocyte-macrophage colony-stimulating factor, IP-10, and tumor necrosis factor (TNF) α; and lower baseline concentration of eotaxin-1, interleukin (IL)-5, and IL-8 were associated with improved DME. Cytokine changes were associated with IVR-induced DME reduction. In zone1, lower concentration of IP-10 compared to baseline or higher concentration of macrophage inflammatory protein (MIP) -α, and in zone 2, lower concentration of IL-5 compared to baseline, IL-8, and IP-10 or higher concentration of eotaxin-1 and MIP-1β were associated with improved DME.

**Conclusions:**

These findings suggest that ranibizumab affects the concentration of cytokines in aqueous humor. Various cytokines contribute to a decrease in retinal thickness, both in the center of the macula and in a larger area of the retina.

## Introduction

Diabetic macular edema (DME) is a complication of diabetic retinopathy that can cause severe visual impairment [1]. Current treatment options for DME include conventional retinal photocoagulation, intravitreal injections of triamcinolone, and vitreous surgery. More recently, anti-angiogenic therapies have been developed. Recent reports have shown that intravitreal injection of bevacizumab (Avastin^®^; Genentech Inc., San Francisco, CA, USA), a full-length humanized monoclonal anti- vascular endothelial growth factor (VEGF) antibody fragment (Fab) specifically designed for ocular use, effectively reduces DME [2,3].

Of all inflammatory and angiogenic cytokines and chemokines involved in the development of DME, vascular endothelial growth factor (VEGF) plays a major role in increasing intraretinal vascular permeability from capillary vessels [4,5,6]. Recent studies have shown that interleukin (IL)-1, IL-6, IL-8, and monocyte chemotactic protein (MCP)-1, are elevated in both the vitreous and aqueous humor of patients with diabetic retinopathy [7,8,9,10,11,12]. Bevacizumab binds to VEGF-A with high affinity, inhibits multiple isoforms of VEGF-A, and has minimal systemic exposure after intravitreal injection [2,12,13]. However, the effect of bevacizumab and other anti-VEGF agents in intraocular cytokine concentration is still contradictory [10,12,14,15].

Three patterns have been described in DME: diffuse retinal thickening (DRT), cystoid macular edema (CME), and serous retinal detachment (SRD). Intravitreal bevacizumab (IVB) inhibits all three types of structural changes, especially DRT [16,17]. Macular retinal thickness can be quantified using optical coherence tomography (OCT). According to the Diabetic Retinopathy Clinical Research Network, center-involved DME is defined as central subfield thickening of at least 250 μm within a 1-mm radius on a Stratus OCT (or a value equivalent in spectral domain OCT [SD-OCT]) and is an indication for treatment. Detailed mapping of macular retinal thickness provides an opportunity to monitor functional changes associated with treatment. However, there have been no quantitative studies on the effects of intravitreal injections of anti-angiogenic agents in DME involving the outer layer of the macula.

We focused on the actions of ranibizumab—an anti-VEGF agent that has been derived from bevacizumab, and compared the cytokine concentration in aqueous humor before and after intravitreal injection of ranibizumab (IVR) in patients with DME. The present author reported previously that eotaxin-1 concentration was significantly decreased in aqueous humor samples after IVR, and that IL-6 concentration also tended to decrease after IVR [18]. In this report, we carried out a further detailed study, and compared the cytokine concentration in aqueous humor before and after IVR in patients with DME. Furthermore, we classified DME based on OCT findings, and determined the changes in cytokine concentration according to type of DME.

## Methods

### Study subjects

This study was performed at The University of Tokyo Hospital (Tokyo, Japan) between July 2014 and January 2015, and was conducted prospectively for patients who received intravitreous ranibizumab (IVR) for DME. Subjects were 12 patients (13 eyes; Table 1). Each eye received two IVR injections with a 1 month interval, and aqueous humor samples were withdrawn at the time of each injection. This study was approved by the Institutional Review Board of The University of Tokyo’s Clinical Research Ethics Committee (#10443), and written informed consent was obtained from all participants prior to initiating the study.

**Table 1:** Baseline clinical characteristics of the patients with diabetic macular edema.

Characteristic	
No. of eyes	13
Sex(male/ female)	5/7
Mean age of patients, y (mean ± SD)	62.5±11.9
Glycated hemoglobin, % (mean ± SD)	6.95±1.3
Treatment	
Oral hypoglycemic agent, n (%)	12 (100)
Insulin, n (%)	3 (25.0)
Hypertension, n (%)	3 (25.0)
Dyslipidemia, n (%)	3 (25.0)
eGFR (n = 11)	64.4±25.6
BCVA (logMAR)	0.47 ± 0.25
CMT of zone1,μm (mean ± SD)	570±109.8
Phakic lens status, n (%)	11 (84.6)
Posterior vitreous detachment, n (%)	4 (30.8)
Stage of diabetic retinopathy, n (%)	
Moderate non-PDR	2 (15.4)
Severe non-PDR	8 (61.5)
PDR	3 (23.1)
History of focal photocoagulation, n(%)	3 (23.1)
History of panretinal photocoagulation, n (%)	7 (53.8)

BCVA = best corrected visual acuity, CMT = central macular thickness, eGFR = estimated glomerular filtration rate, PDR = proliferative diabetic retinopathy, n = number, SD = standard deviation.

Inclusion criteria were as follows: (1) age >20 years; (2) central macular thickness (CMT) ≥250 μm as documented on OCT; (3) no previous IVB or IVR; and (4) a scheduled second IVR performed 1 month after the initial IVR. Exclusion criteria were as follows: (1) previous vitreous surgery; (2) previous cataract surgery within 4 months; (3) previous retinal photocoagulation within 6 months; (4) previous IVB, IVR, or intravitreal injection of triamcinolone acetonide (including subtenon triamcinolone acetonide within 6 months); (5) no scheduled second IVR performed 1 month after the initial IVR; and (6) previous treatment for glaucoma or ocular hypertension. All patients underwent a comprehensive ophthalmic examination, included best-corrected visual acuity, slit-lamp examination, fundus examination using an indirect fundus examination, and intraocular pressure measurements. The evaluation of diabetic retinopathy stage was carried out with the international severity classification from indirect fundus examination findings.

### Measurement and classification of DME

**Fig 1:**
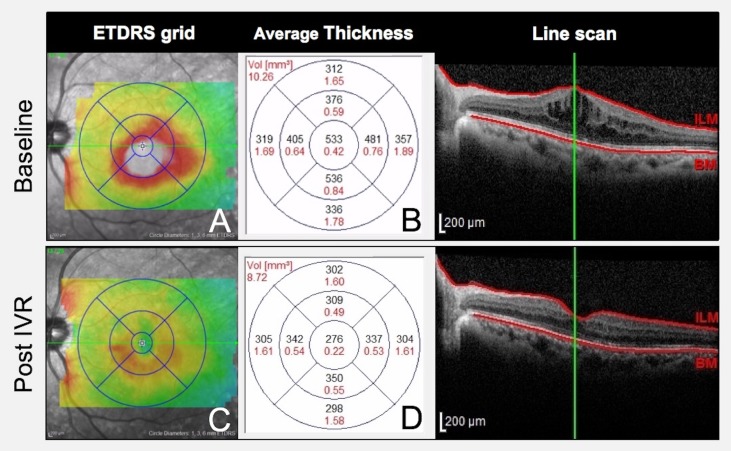
Measurement and evaluation of DME at baseline and post-IVR. OCT findings at baseline and 1 month post-IVR. (A-D) Macular thickness was measured on OCT images using thickness-map mode with an ETDRS 9-zone grid. (B,D) Thickness-map showing the average retinal thickness (μm) in each grid, which projects to the fundus of the eye.

DME was evaluated at baseline and 1 month post-IVR. Macular thickness was measured on a SPECTRALIS^®^ OCT (Heidelberg Engineering GmbH, Dossenheim, Germany) using thickness-map mode with an Early Treatment Diabetic Retinopathy Study (ETDRS) grid (Fig 1A–1D) and the average retinal thickness (μm) on each grid (Fig 1B and 1D). This ETDRS grid was used to demarcate the nine zones limited by solid circles of 1, 3, and 6 mm of diameter centered on the fovea and radial lines, which divide the area within the grid into nine areas of the OCT image projected to the fundus of the eye.

**Fig 2:**
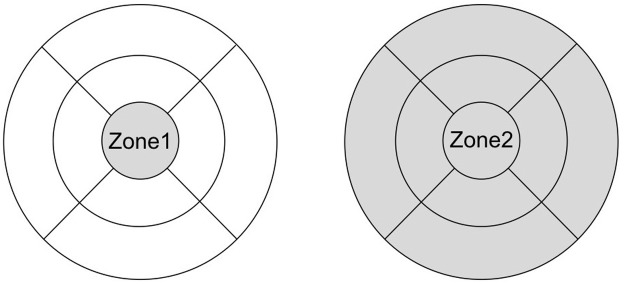
Classification of DME. DME was categorized according to extent and form. The ETDRS grid was divided into two zones: the central circle with a diameter of 1 mm (zone 1); and the outer circle with a diameter of 6 mm (zone 2). Zone 2 allowed the evaluation of larger areas of macular edema. In addition, macular edema was also defined as diffuse if retinal thickness was at least ±30% beyond the reference thickness of zone 1; otherwise, it was defined as non-diffuse.

The extent of macular edema was evaluated based on two zones defined using the ETDRS grid: the central circle with a diameter of 1 mm (zone1); and the outer circle with a diameter of 6 mm (zone 2) (Fig 1). Zone 2 allowed the evaluation of larger areas of macular edema (Fig 2). The mean retinal thickness in zone 1 decreased from 533 μm at baseline to 276 μm post-IVR; the mean retinal thickness of all nine grids, in zone 2 decreased from 406 μm at baseline to 314 μm post-IVR. In zone 1 and zone 2, the effect of IVR was evaluated by the ratio of retinal thickness between baseline and post-IVR.

In addition, macular edema was classified into two types according to form: diffuse (retinal thickness at least ±30% above the reference thickness of zone 1) and non-diffuse (all other cases). Furthermore, macular edema was classified according to the presence or absence of SRD on line-scan OCT (scanning in the horizontal and vertical meridians through the fovea; Fig 1): SRD (presence of any type of SRD) and non-SRD (absence of SRD).

### Intravitreal injection technique and sample collection

After disinfection and draping, 0.05 mL (0.5 mg) of ranibizumab was injected into the vitreous cavity using a 30-gauge needle at a distance of 3.0–4.0 mm from the limbus. A total of 26 undiluted aqueous humor samples (100–200 mL) were collected through a limbal paracentesis site using a 30-gauge needle with a tuberculin syringe from 13 eyes of 12 patients while receiving IVR for DME. Aqueous humor aspiration reduces intraocular pressure after intravitreal injection. All injections and sample collections were performed using a standard sterilization procedure in the operating room. Samples of aqueous humor were collected into sterile tubes at the time of IVR and were rapidly stored at -80°C until analysis.

### Measurement of cytokine concentration by multiplex analysis

Inflammatory cytokine concentration was measured in aqueous samples obtained at baseline and 1 month post-IVR. A multiplexed bead-based immunoassay was used to measure 36 cytokines and chemokines in aqueous humor samples. We used the Luminex^®^100 multiplex array assay (Luminex Corporation, Austin, TX) along with the Bio-Plex^™^ 200 System (Bio-Rad Laboratories Inc.) and bead panel kit (Milliplex^®^ Map Kit Human Cytokine/Chemokine Magnetic Bead Panel, Millipore, cat. #HCYTMAG-60K-PX37; Billerica, MA, USA).

**Fig 3:**
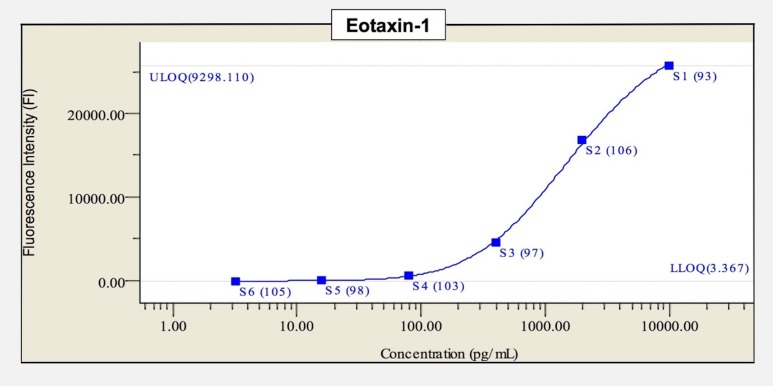
Measurement method of cytokine concentration (the example of eotaxin-1). After plotting the standard curve of eotaxin-1, the fluorescence intensity (FI) of each sample was measured, and the value read from the standard curve was defined as the concentration. S1—S6 are standard samples. S1[9298.11 (concentration), 25760.9 (FI)], S6[3.37 (concentration), 21.9 (FI)]. The detection limit of eotaxin-1 was 3.367 pg/mL. LLOQ = lower limit of quantification ULOQ = upper limit of quantification.

Standard curves for each cytokine were generated from reference standards supplied with the kit. Fig 3 shows the measurement method for eotaxin-1. After plotting the standard curve, the fluorescence intensity of each sample was measured, and the value read from the standard curve was defined as the concentration. Data were analyzed by using Bio-Plex Manager^™^ Software Version 6.0 (Bio-Rad Laboratories Inc.). Cytokine measurements that fell below the limit of detection were deemed as impracticable measurements.

### Outcome measures

The following outcome measures were evaluated: comparison of cytokine concentrations in the aqueous humor between baseline and 1 month post-IVR; baseline cytokine concentration in zone1 and zone2; cytokine changes from baseline to 1 month post-IVR in zone1 and zone2; and cytokine concentration and therapeutic effects of IVR per type of DME.

### Statistical analysis

All statistical analyses were performed with EZR (Saitama Medical Center, Jichi Medical University, Saitama, Japan), a graphical user interface for R (The R Foundation for Statistical Computing, Vienna, Austria). More precisely, it is a modified version of R commander designed to add statistical functions frequently used in biostatistics [19].

Data are presented as mean ± standard deviation. We performed paired t-test to compare the concentration of each cytokine in aqueous humor between baseline and 1 month post-IVR, and multiple regression analysis (Akaike’s information criterion) to establish a relationship between IVR-induced DME reduction and the baseline cytokine concentration, and between reduced DME and changes in cytokine concentration. We performed the Mann–Whitney U-test to compare cytokine concentration among DME types and groups, and to evaluate IVR-induced DME reduction. *P* values <0.05 were considered to indicate a statistically significant difference.

## Results

A total of 26 aqueous humor samples were obtained from 13 eyes performing initial and second IVR. Only those samples with measurable concentration of cytokines and chemokines at baseline and 1 month post-IVR were included in the analysis. Baseline cytokine concentration and cytokine concentration per type of DME were measured for the following molecules: eotaxin-1, granulocyte-macrophage colony-stimulating factor (GM-CSF), IL-1Ra, IL-3, IL-5, IL-8, IP-10, MCP-1, monocyte inflammatory protein (MIP)-1α, MIP-1β, and tumor necrosis factor (TNF)-α. Cytokine changes 1 month post-IVR were determined for eotaxin-1, IL-1Ra, IL-5, IL-8, IP-10, MCP-1, MIP-1α, MIP-1β, and TNF-α.

Among the 36 cytokines measured, the following had low detected levels in all samples or were detected in an insufficient number of samples for comparative study: epidermal growth factor, fibroblast growth factor-2, transforming growth factor-α, granulocyte colony-stimulating factor, fms-related tyrosine kinase-3L, fractalkine, interferon (INF) -α2, INF-γ, growth-regulated oncogene, IL-10, MCP-3, IL-12p40, macrophage-derived chemokine, IL-12p70, IL-13, IL-15, IL-17A, IL-1α, IL-9, IL-1β, IL-2, IL-4, IL-6, IL-7, and TNF-β.

### Changes of aqueous humor concentration

**Fig 4:**
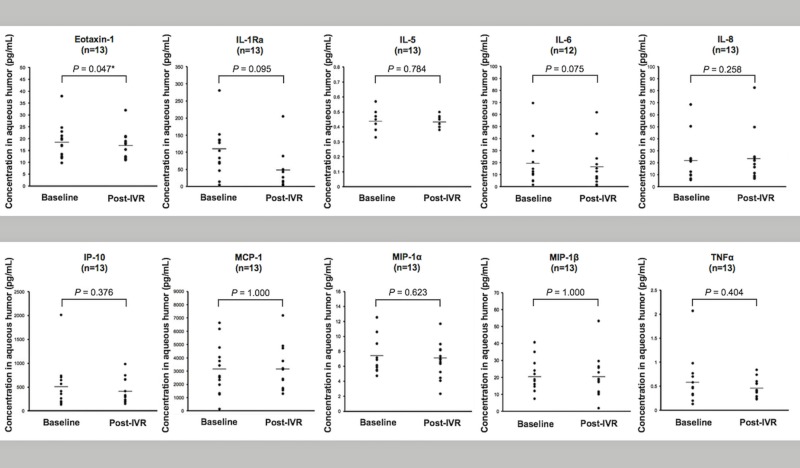
Changes in aqueous humor cytokine concentrations post-IVR. Changes in aqueous humor cytokine concentrations in the 13 eyes between baseline and 1 month post-IVR are shown (IL-6 review of 12 eyes). Eotaxin-1 concentration decreased significantly in aqueous humor samples after IVR (n = 13; *P* = 0.047). IL-6 concentration also tended to decrease after IVR (n = 12; *P* = 0.075).

Eotaxin-1 concentration in aqueous humor samples decreased significantly after IVR (n = 13; *P* = 0.047, paired t-test). In addition, IL-6 concentration also tended to decrease after IVR (n = 12; *P* = 0.075, paired t-test) (Fig 4).

### Baseline cytokine concentration is associated with IVR-induced DME reduction

In zone 1, higher baseline concentration of IP-10 was associated with improved DME (*P* = 0.045). In zone 2, higher baseline concentration of GM-CSF (*P* = 0.027), IP-10 (*P* = 0.015), and TNFα (*P* = 0.025); and lower baseline concentration of eotaxin-1 (*P* = 0.019), IL-5 (*P* = 0.037), and IL-8 (*P* = 0.037) was associated with improved DME (Table 2).

**Table 2:** Relationship between baseline cytokine concentration and IVR-Induced DME reduction (n = 13).

Zone 1			Zone 2		
Variable	Estimate	P value*	Variable	Estimate	P value*
Eotaxin-1	0.13	0.063	Eotaxin-1	-0.09	**0.019**
GM-CSF	-0.39	0.096	GM-CSF	-0.31	**0.027**
IL-3	0.21	0.239	IL-3	0.16	0.066
IL-5	5.38	0.107	IL-5	4.22	**0.037**
IL-8	0.04	0.117	IL-8	0.02	**0.037**
IP-10	-0.001	**0.045**	IP-10	-0.0007	**0.015**
MIP-1α	-0.12	0.324	MCP-1	0.00002	0.382
MIP-1β	-0.26	0.233	MIP-1α	-0.10	0.072
TNFα	-1.94	0.083	MIP-1β	0.02	0.081
			TNFα	-1.33	**0.025**

DME = diabetic macular edema, GM-CSF = granulocyte-macrophage colony-stimulating factor, IL = interleukin, IP = interferon-induced protein, MIP = monocyte inflammatory protein, TNF = tumor necrosis factor.

* Akaike's Information Criterion, bold fond statistical significance (<0.05).

### Cytokine changes are associated with IVR-induced DME reduction

Next, we investigated the relationship between cytokine changes from baseline to 1 month post-IVR and IVR-induced DME reduction. In zone1, lower concentration of IP-10 compared to baseline (*P* = 0.013) or higher concentration of MIP-α (*P* = 0.015) was associated with improved DME. In zone2, lower concentration of IL-5 compared to baseline (*P* = 0.010), IL-8 (*P* = 0.049), and IP-10 (*P* = 0.012) or higher concentration of eotaxin-1 (*P* = 0.031) and MIP-1β (*P* = 0.016) were associated with improved DME (Table 3).

**Table 3:** Relationship between changes in cytokine concentration and changes in macular thickness before and after IVR (n = 13).

Zone 1			Zone 2		
Variable	Estimate	P value*	Variable	Estimate	P value*
IL-5	0.50	0.101	Eotaxin-1	-0.94	**0.031**
IL-8	0.30	0.200	IL-5	0.46	**0.010**
IP-10	0.61	**0.013**	IL-8	0.57	**0.049**
MCP-1	-0.02	0.181	IP-10	0.30	**0.012**
MIP-1α	-0.53	**0.015**	MIP-1β	-0.30	**0.016**
			TNFα	0.01	0.275

DME = diabetic macular edema, IL = interleukin, IP = interferon-induced protein, MCP = monocyte chemotactic protein, MIP = monocyte inflammatory protein, TNF = tumor necrosis factor.

* Akaike's Information Criterion, bold fond indicates statistically significance (<0.05).

### Cytokine concentration and therapeutic effects of IVR are similar between types of DME

We classified DME into diffuse (4/13 eyes [31%]) and non-diffuse (9/13 eyes [69%]). The concentrations of various cytokines in the aqueous humor were compared between the two types. However, there were no differences in the concentration of eotaxin-1, GM-CSF, IL-3, IL-5, IL-8, IP-10, MCP-1, MIP-1α, MIP-1β, and TNFα (Mann–Whitney U-test). The therapeutic effects of IVR were also compared, but there were no differences in the efficacy of IVR between the two types in zone1 (*P* = 0.940) and zone2 (*P* = 0.825).

We also classified DME into SRD (5/13 eyes [38%]) and non-SRD (8/13 eyes [62%]). The SRD group tended to have higher concentration of IL-1Ra (Mann–Whitney U-test). However, IVR-induced improvement of retinal thickness was comparable between SRD and non-SRD, and there were no differences per zone (Table 4).

**Table 4:** Cytokine concentrations according to DME type (n = 13).

	Diffuse vs. non-diffuse			SRD vs. non-SRD		
	Diffuse (n = 4)	Non-diffuse (n = 9)	P value*	SRD (n = 5)	Non-SRD (n = 8)	P value*
Eotaxin-1 (pg/mL)	19.9 ± 5.7	18.0 ± 8.4	0.503	21.6 ± 11.2	16.7 ± 3.7	0.435
GM-CSF (pg/mL)	1.5 ± 1.1	1.8 ± 1.1	0.587	1.7 ± 1.4	1.7 ± 0.9	0.713
IL-1Ra (pg/mL)	88.8 ± 65.9	97.0 ± 83.4	1.000	148.1 ± 84.4	60.9 ± 49.1	0.065
IL-3 (pg/mL)	1.7 ± 0.9	2.6 ± 1.0	0.199	2.4 ± 1.3	2.3 ± 1.0	0.622
IL-5 (pg/mL)	0.4 ± 0.1	0.4 ± 0.1	0.876	0.5 ± 0.1	0.4 ± 0.1	0.414
IL-8 (pg/mL)	19.0 ± 6.1	22.9 ± 22.3	0.825	26.5 ± 24.4	18.6 ± 14.9	0.524
IP-10 ((pg/mL)	870.6 ± 778.8	342.0 ± 233.3	0.148	602.8 ± 790.8	443.4 ± 253.4	0.943
MCP-1 (pg/mL)	3295.5 ± 1087.2	3099.6 ± 2245.5	0.710	2556.4 ± 1037.5	3537.1 ± 2290.6	0.354
MIP-1α (pg/mL)	6.8 ± 2.6	7.1 ± 2.4	0.414	7.8 ± 3.6	6.5 ± 1.2	0.724
MIP-1β (pg/mL)	21.2 ± 10.0	19.9 ± 9.9	0.877	21.4 ± 15.2	19.6 ± 4.7	0.608
TNFα (pg/mL)	0.4 ± 0.3	0.7 ± 0.6	0.246	0.8 ± 0.7	0.4 ± 0.3	0.212

GM-CSF = granulocyte-macrophage colony-stimulating factor, IL = interleukin, IP = interferon-induced protein, MCP = monocyte chemotactic protein, MIP = monocyte inflammatory protein, TNF = tumor necrosis factor, SRD = serous retinal detachment, n = number

*Mann–Whitney U-test

## Discussion

Higher baseline concentration of IP-10 was associated with improved DME in zone1 and zone2. However, as the concentration of IP-10 decreased post-IVR, DME also improved. The concentration of IP-10 in aqueous humor are elevated in patients with diabetic retinopathy [11,12], and are significantly higher in each of the three patterns of DME compared to control [11]. IP-10 (*CXCL10*) was initially known as an early, transiently expressed, interferon (IFN) γ-inducible gene in a histiocytic lymphoma cell line with monocytic characteristics [20]. Recently, IP-10 has been reported to inhibit angiogenesis [21,22,23]. Liu et al. showed that IP-10 inhibits angiogenesis by reducing VEGF concentration in mice with alkali-induced corneal neovascularization [23]. This study showed that treatment of DME was more effective in eyes with higher baseline concentration of IP-10, but that a decrease of IP-10 concentration post-IVR contributed to a decreased of outer retinal thickness, not only central retinal thickness.

The opposite was observed with eotaxin-1. Lower baseline concentration of eotaxin-1 was associated with improved DME in zone2. However, as the concentration of eotaxin-1 increased post-IVR, DME also improved. eotaxin-1 (known as CCL11) is acting on the CCR3 receptor. CCR3 binds many chemokines (e.g., CCL5/7/8/13/15/28) in addition to CCL11 (eotaxin-1)/CCL24 (eotaxin-2)/CCL26 (eotaxin-3). There are several reports on eotaxin-1 and age-related macular degeneration (AMD). The signaling downstream CCR and VEGF is particularly important to choroidal neovascular neovascularization (CNV) in AMD [24], particularly the association with VEGFR-2, which plays a key role in the proliferation of vascular endothelial cells [25]. Furthermore, another report mentioned that blockade of CCR3 is more effective than VEGF neutralization in reducing CNV [24]. Although the pathogenesis of AMD and DME may differ in terms of a molecular interaction between VEGFR-2 and CCR3, it also considers an interaction between CCR3 and VEGF-A, which binds to VEGFR-2. This supports the relationship between eotaxin-1 and improvement of DME.

As baseline concentration of IL-5 and IL-8 were low, retinal thickness of zone2 was reduced by IVR. IVR-induced reduction of IL-5 and IL-8 was also associated with decreased retinal thickness. IL-5 was identified as a T-cell replacing factor (TRF) in a study of antibody production by mycobacterium tuberculosis (so-called purified protein derivatives) [26]. IL-5, along with IL-4, is a known marker of Th2 differentiation. It also induces eosinophilia upon parasitic infection or allergic inflammation. IL-8 is known as a neutrophil chemotactic factor and T-cell activator in the innate immune system. It was reported that IL-8 is increased in the vitreous fluid in retinal artery occlusion and proliferative diabetic retinopathy [8], and aqueous concentration of IL-8 have been shown to increase with progression of diabetic retinopathy from mild to severe non-proliferative diabetic retinopathy [27]. According to previous reports, IL-8 is an indicator of DMR activity. Our findings suggest that IL-8 could also serve as an indicator of IVR-induced DME reduction.

As baseline concentration of TNFα and GM-CSF were higher, retinal thickness decreased. TNFα is a factor derived from a host to induce necrosis of the tumor, which is produced in response to endotoxins. TNFα regulates a variety of the biological functions; disturbed TNFα signaling is related to various diseases. Although TNFα is produced primarily by activated macrophages, its expression is found in a wide range of cells [28].

GM-CSF plays an important role in neutrophil induction and macrophage production and activation. According to previous studies, GM-CSF concentration are decreased in the aqueous humor of patients with diabetic retinopathy. TNF-α has been shown to regulate production in retinal pigment epithelial cells. However, there is limited understanding of the role of GM-CSF role in the pathogenesis of diabetic retinopathy [29].

TNFα and GM-CSF have broad functions, and there is a close connection between these two cytokines. Although we cannot conclude their role in diabetic retinopathy, the concentration of these cytokines changed with IVR. Hence, these cytokines are likely involved in DME.

As a result of the examination 2, the zone1 showed that as concentration of MIP-α was high, and the zone2 showed that MIP-1β were low, macular edema decreased. Both MIP-1α and MIP1-β show the migration activity to human neutrophils. MIP-1β has also been reported in proliferative diabetic retinopathy, and has been suggested to play a role in attracting and activating leukocytes in inflammation [30,31,32]. In our study, although the concentration of MIP-1α and MIP1-β elevated in case DME was improved by IVR, there was also possibility that its improvement had been affected by diabetic retinopathy itself, not by IVR.

Although previous studies have been conducted in order to find out whether anti-VEGF agents had effectiveness on inflammatory cytokines except VEGF, there have been conflicting reports exist. This study showed as a result of the examination 2 that eotaxin-1, IP-10, MIP-α, IL-5, IL-8, IP-10, and MIP-1β was relevant to the effectiveness of IVR in reducing macular edema. IL-6, IP-10, MCP-1, PDGF-AA, and VEGF were significantly decreased in triamcinolone-treated eyes, whereas only VEGF was decreased in IVB eyes [12]. Another previous report also demonstrated that effectiveness of IVB for DME had no effect on IL-6, IL-8, and MCP-1 [10]. In this study, however, the concentration of cytokines in aqueous humor samples collected during IVB were compared native IVB group with group of previously IVB received. The group with a history of IVB had received treatment in average 9.6 ± 1.4 weeks (range, 8–12 weeks) prior to this study. Therefore, it is possible that bevacizumab attenuated the effects seen in aqueous cytokines.

In contrast, there have been reports that anti-VEGF agents affect inflammatory cytokines. Aqueous humor samples were taken at the time of IVB pretreatment, and the concentration of 20 different cytokines was determined. This result showed that the concentration of IL-8 and TGF-β2 increased after IVB [14]. Moreover, IL-8 concentration also increases after IVR [15].

In these two reports, aqueous humor was obtained from patients with proliferative diabetic retinopathy, which had nonclearing vitreous hemorrhage and/or tractional retinal detachment requiring pars plana vitrectomy (PPV), and the concentration of cytokines samples in aqueous humor were compared at the time of IVB performed as pre-treatment approximately 1 week before PPV during the surgery. In short, these studies investigated proliferative diabetic retinopathy cases and were not limited to DME, and measured the concentration of cytokines within 1 week after the administration of anti-VEGF agents. Clinically, administering intravitreal anti-VEGF agents for DME every 4 weeks is common; therefore, the 1-week time point was probably too early. Furthermore, the authors restricted their analyses to placental growth factor (PIGF), IL-8, and TGF-β2; other cytokines were not compared. According to our study, by considering a wider range of cytokines, we were able to obtain further knowledge.

Although DME was classified into two groups that are diffuse type or non-diffuse type to determined significant differences in concentration of cytokines, there were no differences between two groups. In addition, a previous report demonstrated that diffuse DME is caused by severe and diffuse leakage from damaged capillaries involved with damage to the blood–retinal barrier [33]. Although anti-VEGF agents were more effective in diffuse DME than in CME because they reduced vascular permeability in retina, in this study there were was no differences in the effectiveness of IVR.

In agreement with previous studies, IL-1Ra—a receptor to pro-inflammatory IL-1—was upregulated in the SRD group. However, there were no differences between groups in the improvement rate of retinal thickness by IVR. According to previous reports, the pathogenesis of CME and SRD is related to prostaglandins and inflammatory cytokines as well as VEGF [34,35]. IVR had similar effects regardless of DME type. However, our sample size was small. A larger study is warranted in future.

In summary, inflammatory cytokine concentration in aqueous humor were comprehensively measured to determine the effectiveness of IVR in reducing DME. As our new findings, we evaluated DME in more extensive scope with retinal mapping on OCT rather than only in the fovea of DME, as previously reported. We found that each cytokines influenced on decreasing of retinal thickness by IVR, and both center of macula and in a wider range of retina were affected by different cytokines. Among the cytokines affected by IVR, low IP-10 concentration was important to improve DME. Furthermore, this study also suggested that effectiveness of IVR in reducing DME was related to eotaxin-1, IL-8, TNFα, and GM-CSF. Although DME pattern was classified into diffuse and non-diffuse based on a thickness-map mode installed in the OCT, there was no significant difference between these two types. We also compared cytokine concentration of DME between SRD and non-SRD; IL-1Ra concentration tended to be higher in the SRD group. Nevertheless, this study supports that variation of cytokine concentration is associated with IVR-induced DME reduction.
